# Feature recess-time sports activities as a school-based intervention to improve fitness in rural Chinese youth

**DOI:** 10.1371/journal.pone.0337716

**Published:** 2026-01-22

**Authors:** Xiao Hua Huang, Xiao Yu Huang, Fadzilah Abd Rahman

**Affiliations:** 1 Xiayi County Senior High School, Shangqiu, Henan, China; 2 Faculty of Education & Liberal Studies, City University, Kuala Lumpur, Malaysia; 3 Faculty of Social Sciences and Liberal Arts, UCSI University, Kuala Lumpur, Malaysia; 4 Department of Professional and Continuing Education, School of Education, Sunway University, Petaling Jaya, Malaysia; Shahid Chamran University of Ahvaz, IRAN, ISLAMIC REPUBLIC OF

## Abstract

**Background:**

China’s national school health policies face persistent implementation gaps, particularly in rural high schools prioritizing Gaokao. National surveys (2010–2019) documented alarming fitness declines: 50-m sprint speeds decreased (+0.3s boys; + 0.4s girls), pull-ups/sit-ups fell 29%/20%, and standing jumps shortened 6–7 cm.

**Methods:**

A 16-week cluster quasi-experiment assigned intact rural high school classes (N = 98; age = 16.35 ± 0.48years) to: • Experimental (n = 50): Feature Recess-Time Sports Activities (FRTSA; 5x30-min/week). • Control (n = 48): Standard supervised running. Blinded assessors conducted the National Student Physical Health Standard tests.

**Results:**

FRTSA elicited significant improvements versus control: • Speed: 50-m sprint (*p < 0.001,* η^*2*^ = 0.18, *d* = 0.75). • Explosive Power: Standing jump (*p* = 0.022, η^*2*^ = 0.05, *d* = 0.41). • Flexibility: Sit-and-reach (*p* < 0.001, η^*2*^ = 0.12, *d* = 0.60). • Strength: Male pull-ups (*p* = 0.030, d = 0.41); female sit-ups (*p = 0.029, d = 0.45*). No endurance benefits emerged (1000m/800m: all *p* > 0.05, *d* ≤ 0.18).

**Conclusion:**

FRTSA is effective in enhancing speed, explosive power, flexibility, and strength, supporting policy integration of structured activity programs in rural schools.

## Introduction

Physical fitness (PF) is a fundamental determinant of adolescent health [1,2], with adolescence constituting a critical window for establishing lifelong physical activity behaviors [3–5]. Robust evidence connects PF to: (a) cardiometabolic health indicators [6,7], (b) academic achievement [8], and (c) psychological well-being [9,10]. Although China’s *Guidelines for Teaching Reform in Sports and Health* (Trial) [11] emphasize youth health as a national priority, rural resource disparities – including physical education (*PE*) teacher shortages [12], equipment deficits [13], and inadequate facilities [14] – result in significantly lower *PF* levels among rural versus urban adolescents (*p* < 0.05) [15, 16].

To address these challenges, recess-time sports activities (RTSA) provide a promising, low-cost strategy to increase daily physical activity [17–20]. In this study, we introduce “Featured Recess-Time Sports Activities” (FRTSA)—a structured, theory-informed intervention designed to enhance engagement and fitness outcomes in resource-constrained settings. Unlike traditional RTSA, which is typically unstructured and student-led, FRTSA integrates guided instruction, progressive skill development, and music-paced routines to promote sustained moderate-to-vigorous physical activity (MVPA) [21].

The FRTSA model was developed using the ADDIE framework (Analysis, Design, Development, Implementation, Evaluation) to ensure systematic planning and iterative refinement [22]. It incorporates cooperative learning principles—emphasizing peer interaction, group goals, and shared responsibility—and scaffolding techniques, where activities are sequenced from basic to advanced with teacher support gradually reduced over time [23,24]. This theoretical foundation supports both motor skill acquisition and social-emotional development.

Building on China’s Sunshine Sports initiative (2007), FRTSA features: (1) scientific design (integrating PE guidelines and exercise physiology), (2) safety protocols (WHO-approved risk mitigation), (3) diversified modalities (≥1 activity type/session), and (4) adaptive flexibility (aligned with developmental needs and facility conditions) [25–27]. Although endorsed internationally [28,29], rural implementations face barriers such as spatial constraints (2.3 ± 0.8 m^2^/student vs. national ≥5.0 m^2^ standard) [30,31], equipment deficits [32], and organizational rigidity [33].

Current literature reveals two key gaps: (i) limited exploration of high school RTSA frameworks, and (ii) insufficient research on rural adolescent PF outcomes. To address these, we developed and tested an FRTSA model tailored for rural high schools. Using a dynamic distributed–concentrated approach, the model overcomes space and equipment limitations, offering a scalable solution for underserved populations.

The FRTSA program integrates four evidence-based components: (1) functional music gymnastics (coordination), (2) fun snake run (aerobic endurance), (3) figure skipping rope (gross motor skills), and (4) yoga-based relaxation (emotional regulation). Delivered over 16 weeks in a music-paced format (WHO standards, 2022) [34], FRTSA aims to optimize both physiological gains and student engagement during recess. This quasi-experimental study evaluated its efficacy in improving five PF domains: speed (50-m sprint), explosive power (standing long jump), flexibility (sit-and-reach), strength (pull-ups/sit-ups), and endurance (800/1000-m run). We hypothesized greater PF improvements in the experimental group (α = 0.05, two-tailed), providing an evidence-based strategy to reduce urban–rural fitness disparities through scalable school-based programming.

## Materials and methods

### Study design

Given the ethical constraints of randomizing intact school classes [35], we employed a two-arm quasi-experimental design to evaluate the *feature recess-time sports activities* (*FRTSA*) effects on physical fitness among rural Chinese high-school freshmen. The study protocol received ethical approval from Henan Normal University’s Institutional Review Board and strictly adhered to *Belmont Report* principles [36], with particular attention to: (1) respect for persons (written informed consent from all participants), (2) beneficence (risk-benefit analysis conducted prior to intervention), and (3) justice (equitable selection of participant schools).

While cluster randomization at the school level is considered optimal for minimizing contamination in school-based interventions, logistical and administrative constraints limited our ability to implement multi-school randomization. Instead, we conducted class-level randomization within a single school, which allowed for close supervision and consistent implementation while preserving classroom integrity. Sensitivity analyses were conducted to assess potential clustering effects, and intraclass correlation (ICC) was found to be low (< 0.05), suggesting minimal within-class dependency.

### Study population and sampling

#### Paragraph 1: Sampling framework.

This study employed a multistage sampling approach to recruit high-school freshmen from Xiayi County, Henan Province, comprising: (1) stratified random selection of Henan from China’s 34 provincial divisions, (2) random selection of Shangqiu from Henan’s 17 prefecture-level cities, (3) random selection of Xiayi from Shangqiu’s six county-level districts, and (4) purposive selection of Xiayi County X High School (representative rural institution) from four local schools based on enrollment size and facility availability.

#### Paragraph 2: Participant recruitment.

Participants were composed of two randomly – selected Grade 1 classes (N = 98). All participants met the inclusion criteria:

Age between 16 and 17 years
No diagnosed physical impairments
Obtained parental consent and student assent

The experimental group (n = 50; 26 males, 24 females; mean age = 16.4 ± 0.5 years) received FRTSA, whereas the control group (n = 48; 25 males, 23 females; mean age = 16.3 ± 0.5 years) continued with the standard RTSA. Baseline equivalence was confirmed for age (mean difference [MD] = 0.1 years, 95% confidence interval [CI]: [−0.2, 0.4], *p* = 0.76) and sex distribution (χ^2^ = 0.02, *p* = 0.92).

The two classes were selected based on comparable size, gender distribution, and baseline physical fitness levels. One class was assigned to the intervention group and the other to the control group through a random lottery draw conducted by an independent research coordinator. Given the school-based nature of the intervention and the need to preserve classroom integrity, students were not individually randomized but participated as intact class units. This approach balanced feasibility with methodological rigor in a real-world educational setting.

#### Paragraph 3: Sample size justification.

The sample size was determined using GPower 3.1. The following parameters were considered: a medium effect size (d = 0.5, as defined by Cohen in 1988), a significance level (α) of 0.05, and a statistical power of 0.80 [37]. Based on these specifications, the minimum required sample size (N) was calculated to be 92, with 46 participants per group. The final sample size, with N = 98, exceeded this calculated threshold, thus ensuring adequate statistical power for the study.

### Intervention protocol

The 16-week intervention (September–December 2024) consisted of five 30-minute FRTSA sessions per week, integrating functional music gymnastics, fun snake run drills, structured figure skipping rope training, and guided yoga-based relaxation exercises. Pre- and post-intervention assessments were administered based on the China National Student Physical Fitness Standard (CNSPFS, 2014 revision) [38].

### Outcome measurements

Anthropometric and physical fitness assessments were conducted before and after the 16-week intervention, following CNSPFS, 2014 revision (See S1 Appendix). Standardized tests included:

**Speed:** 50-m sprint
**Explosive Power:** Standing long jump
**Flexibility:** Sit-and-reach test
**Strength:** Pull-ups (males)/ 1-min sit-ups (females)
**Endurance**: 1000-m run (males)/ 800-m run (females)

All measurements were conducted simultaneously for male and female students using CNSPFS-certified instruments (Table 1). Students in the experimental group completed their tests at the East Playground, while students in the control group were assessed at the West Playground. To minimize the impact of the endurance events (800-m and 1000-m runs) on subsequent tests, endurance assessments were scheduled on separate days. Except for the speed (50-m sprint) and endurance (800-m/1000-m run) tests, all other physical fitness tests were performed twice, with the best result recorded.

**Table 1:** Measurement items, instruments, and procedures.

Domain	Test Item	Instrument/Equipment	Procedure	Outcome
Body Composition	Height, Weight, BMI	InBody BSM370 (Korea)	Participants stood barefoot on the platform; the system automatically measured height and weight and calculated BMI.	BMI (kg/m^2^)
Speed	50-m Sprint	Signal flag, whistle, stopwatch	Participants sprinted 50 meters at a signal; time was recorded with a stopwatch.	Time (seconds)
Explosive Power	Standing Long Jump	Specialized soft cushion for standing long jump	Participants jumped forward from a standing position; the best distance of two trials was recorded.	Distance (cm)
Flexibility	Sit-and-Reach	Sit-and-reach measurement device and mat	Participants reached forward with extended legs; best of two trials recorded.	Distance (cm)
Strength	Male: Pull-up Test	Intelligent pull-up tester (Huake Zhichuang HK-PU01)	Male participants performed pull-ups with valid repetitions automatically counted by the device.	Repetitions (count)
	Female: One-minute Sit-up Test	Soft cushion for sit-ups, stopwatch	Female participants completed as many correct sit-ups as possible in one minute.	Sit-ups (count)
Endurance	Male: 1000-m Female: 800-m	Starting gun, stopwatch	Participants completed the designated distance run; time was recorded upon finishing.	Time (seconds)

Although the total area of the East and West Playgrounds differed, the designated testing zones were comparable in surface material (poured rubberized concrete), flatness, and available space—all exceeding minimum requirements for each assessment. All tests were conducted outdoors on the same day and during the same time window (9:00–11:00 AM), under clear, windless conditions. No differences in lighting, temperature, or noise interference were observed between groups during testing.

### Physical fitness assessment procedures

#### Body mass index test.

A portable high-precision anthropometric system (InBody BSM370, Korea) was used to assess biometric parameters. Participants stood barefoot on the measurement platform in a standardized anatomical position, ensuring heel alignment with the foot electrodes. After inputting age and gender data, the system automatically measured body weight (kg) and height (m) with medical-grade precision. Body mass index (BMI) was calculated instantly, and results were displayed on an LCD screen.

#### 50-m sprint test.

The 50-m sprint test assessed speed performance using a racetrack, starting flag, whistle, and stopwatch. Participants, wearing sports attire, performed a standardized warm-up and assumed a ready stance behind the starting line. At the starter’s signal, they sprinted toward the finish line while the timekeeper recorded the completion time in seconds (s).

#### Standing long jump test.

The standing long jump test evaluated lower-limb explosive strength and coordination. Prior to testing, participants wore sports shoes and loose-fitting sportswear and completed warm-up exercises. During the test, participants stood with feet shoulder-width apart behind the take-off line, jumped forward with both feet simultaneously, and exited the landing area from the front after landing. Scoring was based on the position of the heels; if misaligned, the rear heel’s position was recorded. Each participant completed two trials, with the best score, measured in centimeters (cm), retained for analysis.

#### Sit-and-reach test.

The sit-and-reach test assessed flexibility, joint mobility, and muscle elasticity. Before testing, participants removed their shoes, wore loose sportswear, and positioned the measuring device against a wall with a soft mat in front. Participants sat with legs fully extended, heels together, toes approximately 30 degrees apart, and feet pressed against the device. With palms facing downward, they reached forward, pushing the sliding marker with their fingertips while maintaining straight knees and continuous contact between legs and mat. Scores were recorded based on the final vernier reading in centimeters (cm).

#### Pull-up test (male students).

Upper-body muscular strength and endurance were assessed using the Huake Zhichuang HK-PU01 intelligent pull-up tester. After a standardized warm-up, male participants grasped the horizontal bar with a shoulder-width overhand grip and fully extended arms. Upon activation of the device, participants performed pull-ups, ensuring that the chin cleared the bar for each valid repetition. The built-in sensors and AI recognition system automatically recorded valid counts and provided real-time feedback. Excessive body movement was prohibited, and a pause exceeding 10 seconds resulted in automatic test termination. Pull-up performance was measured in number of repetitions (count).

#### One-minute sit-up test (female students).

The one-minute sit-up test assessed abdominal and lumbar muscular endurance. Female participants, in pairs, completed the test, with one student stabilizing the ankles while the other performed sit-ups. Participants began in a supine position with hands interlocked behind the head, knees bent at 90°, and feet flat on the ground. On command, participants rose until their elbows touched their knees and returned to the starting position with shoulders contacting the mat. The total number of sit-ups completed in one minute was recorded. Proper breathing technique—exhaling during ascent and inhaling during descent—was required; breath-holding was prohibited.

#### 1000-m/800-m Endurance test.

The 800-meter (female) and 1000-meter (male) run tests evaluated cardiovascular endurance. Participants wore sports attire and completed a standardized warm-up before testing. Students were grouped in sets of eight, and the course was marked by the teacher. Timing commenced at the start signal using a stopwatch, with participants adopting a staggered stance and maintaining rhythmic breathing patterns (e.g., inhaling every two to three steps). The final time, recorded in seconds (s), was documented upon crossing the finish line. Male students were tested first, followed by female students. After completing the test, participants engaged in cool-down exercises such as walking or light stretching to regulate breathing and were encouraged to hydrate.

### Intervention program

The Feature Recess Time Sports Activities (FRTSA) intervention was implemented over 16 weeks, structured into four progressive stages. Weeks 1–4 (basic stage) emphasized the acquisition of fundamental movement skills. Weeks 5–8 (consolidation stage) focused on reinforcing and mastering these skills. Weeks 9–12 (improvement stage I) involved integrating all FRTSA components with musical accompaniment. Finally, weeks 13–16 (improvement stage II) aimed to refine movement proficiency and optimize overall performance. During the same period, the control group participated in a structured, military-style group running program implemented without musical accompaniment and directed by whistle cues from a physical education teacher. The session lasted 30 minutes daily and consisted of two phases: (1) Group Formation Running (25 min), during which students ran in square formations following standardized commands—initiated by a first whistle for double-time jogging and a second for quick-time walking, emphasizing rhythmic uniformity; and (2) Cool-Down and Dismissal (5 min), involving a silent, relaxed group walk followed by orderly return to the classroom led by a student monitor. This routine reflected the standard physical activity practice in many rural Chinese schools and served as the comparative baseline for evaluating the FRTSA intervention.

The FRTSA program incorporated functional music gymnastics, fun snake run, jump rope training, and yoga-based relaxation exercises. These components were designed to systematically enhance students’ flexibility, coordination, endurance, and overall physical fitness. The exercise components and the detailed implementation procedures are provided in Table 2*.*

**Table 2:** Implementation schedule and exercise components.

Week	Session	Exercise	Content Description	Duration (min)
1	1	Functional music gymnastics	Preparatory warm-up	10
1	2	Functional music gymnastics	Upper limb movements	10
1	3	Functional music gymnastics	Chest exercises	10
1	4	Functional music gymnastics	Running and jumping drills	10
1	5	Functional music gymnastics protocol	Full practice session	15
2	6	Fun snake run	Mark and fix student positions, designate lead runners	0
2	7	Fun snake run	Walking simulation of a serpentine route while maintaining spacing	15
2	8	Fun snake run	Command-driven running drill on a serpentine route	15
2	9	Fun snake run	Rhythm-based running drill on a serpentine route	10
2	10	Fun snake run protocol	Full practice session	10
3	11	Figure skipping rope	Parallel-leg single swing jump, cross jump, open-close jump	10
3	12	Figure skipping rope	Lunge jump, leg rotation jump	10
3	13	Figure skipping rope	Alternate foot skip, knee-lift jump	10
3	14	Figure skipping rope	Kick jump, one-minute speed jump	10
3	15	Figure skipping rope protocol	Full practice session	15
4	16	Yoga-based relaxation exercises	Deep breathing and shoulder stretching exercises	10
4	17	Yoga-based relaxation exercises	Chest extension, arm extension, stretching the back of the legs	10
4	18	Yoga-based relaxation exercises	Rear arm stretch, yoga standing extension	10
4	19	Yoga-based relaxation exercises	Front thigh extension, breathing adjustment	10
4	20	Yoga-based relaxation exercises protocol	Full practice session	15
5	21–22	Functional music gymnastics & Fun snake run	Practice under rhythmic commands	10
5	23–25	Functional music gymnastics & Fun snake run	Practice in synchronization with music	15
6	26–27	Fun snake run & Figure skipping rope	Practice under rhythmic commands	10
6	28–30	Fun snake run & Figure skipping rope	Practice in synchronization with music	15
7	31–32	Figure skipping rope & Yoga-based relaxation exercises	Practice under rhythmic commands	10
7	33–35	Figure skipping rope & Yoga-based relaxation exercises	Practice in synchronization with music	15
8	36–37	Functional music gymnastics, Fun snake run & Figure skipping rope	Practice under rhythmic commands	10
8	38–40	Functional music gymnastics, Fun snake run & Figure skipping rope	Practice in synchronization with music	15
9	41–42	Functional music gymnastics, Fun snake run, Figure skipping rope & Yoga-based relaxation exercises	Practice under rhythmic commands	10
9	43–45	Functional music gymnastics, Fun snake run, Figure skipping rope & Yoga-based relaxation exercises	Practice in synchronization with music	15
10	46–48	Functional music gymnastics & Fun snake run	Movement correction practice	10
10	49–50	Functional music gymnastics & Fun snake run	Practice in synchronization with music	10
11	51–53	Figure skipping rope & Yoga-based relaxation exercises	Movement correction practice	10
11	54–55	Figure skipping rope & Yoga-based relaxation exercises	Practice in synchronization with music	10
12–16	56–80	Functional music gymnastics, Fun snake run, Figure skipping rope & Yoga-based relaxation exercises	Full practice session	10

### Participant testing and data collection procedures

Two physical education teachers from Xiayi County Senior High School were first trained in the implementation of the intervention protocol. Subsequently, professional coaches from the Xiayi County Sports Education Bureau conducted standardized tests of participants’ height, weight, 50-meter sprint, pull-ups, 1-minute sit-ups, standing long jump, and sit-ups. The test results were immediately submitted to the research team for baseline evaluation. During the tests, the physical education teachers were responsible for maintaining order and ensuring the safety of the participants. They also led students through warm-up exercises before testing and guided them to each subsequent testing station.

### Program relevance, engagement, and adherence monitoring

Perceived program relevance—reflecting students’ attitudes and motivation—was assessed in the FRTSA group using a 5-item survey at post-intervention. Items addressed enjoyment, perceived physical benefit, and willingness to continue, rated on a 5-point Likert scale (1 = strongly disagree, 5 = strongly agree). Internal consistency was acceptable (α = 0.76), and mean item scores were calculated for descriptive analysis.

Engagement and adherence were systematically monitored through daily attendance records and structured session logs maintained by physical education teachers. Adherence was defined as participation in ≥80% of scheduled sessions (≥ 64 out of 80 sessions over 16 weeks). Students attending fewer than 52 sessions (< 65%) were classified as low adherence and included in sensitivity analyses.

Instructional fidelity was ensured through a dual-layer monitoring system. All teachers received standardized training prior to implementation, covering procedural consistency and safety protocols. Weekly supervisory visits by research staff verified protocol adherence, and any deviations were documented in activity logs. This approach supported consistent delivery of both FRTSA and control conditions.

### Statistical analysis

This study adhered to the CONSORT extension guidelines for non-randomized trials. Although the statistical analysis plan was developed a priori, it was not formally preregistered. All analyses were conducted using *R* version 4.3.2.

Baseline equivalence between the experimental and control groups was assessed using independent-samples *t*-tests for continuous variables and χ^2^ tests for categorical variables, with all comparisons yielding non-significant results (*p > 0.05*). Parametric assumptions were confirmed (Shapiro–Wilk test *W > 0.95*; Levene’s test *p > 0.15*). Within-group changes were analyzed using paired *t*-tests, and effec*t* sizes were reported as Cohen’s *d* with 95% confidence intervals (CIs), classified as small (0.2–0.49), medium (0.5–0.79), and large (≥0.8).

Between-group differences were evaluated using analysis of covariance (ANCOVA) adjusting for baseline scores. To verify the assumption of homogeneity of regression slopes, we tested the interaction between group assignment and baseline fitness scores for each outcome. No significant interactions were observed (all p > 0.05), supporting the validity of the ANCOVA model. Results were presented as adjusted mean differences (ΔΔ) with 95% CIs, partial η^2^ values (interpreted as small ≥0.01, medium ≥0.06, large ≥0.14), and standardized mean differences. Analytical rigor was ensured through bootstrapped CIs (1,000 resamples), outlier screening (excluding <5% of cases), and Holm–Bonferroni corrections for multiple comparisons. All tests were two-tailed with a significance threshold set at α = 0.05. The study was powered (>80%) to detect medium-sized effects (*d = 0.5)*. Complete analysis scripts are available from the authors upon reasonable request.

## Results

### Baseline equivalence between groups

Baseline characteristics were balanced between the experimental and control groups, with no statistically significant differences in age, sex, or BMI (*p > 0.05*; Table 3). Similarly, baseline performance across all physical fitness assessments showed no significant differences between groups (*p > 0.05*; Table 4). The effect sizes were trivial (Cohen’s *d* < 0.5), indicating minimal pre-intervention disparities.

**Table 3:** Baseline demographic and anthropometric characteristics.

Variable	Control Group (n = 48)	Experimental Group (n = 50)	p-value	Test Statistic
Age (years)	16.33 ± 0.48	16.36 ± 0.48	0.759	*t*(96) = 0.31
Gender (Male:Female)	25:23	26:24	0.981	*χ*^2^(1) = 0.001
BMI (kg/m^2^)	22.58 ± 1.97	22.85 ± 1.93	0.570	*t*(96) = 0.57

**Table 4:** Baseline physical fitness test comparisons.

Measure	Control Group (n = 48)	Experimental Group (n = 50)	p-value	Cohen’s d	Test Statistic
50m sprint (s)	8.81 ± 0.71	8.83 ± 0.88	0.902	−0.03	*t*(96) = −0.12
Standing long jump (cm)	208.46 ± 37.54	208.96 ± 37.35	0.947	−0.01	*t*(96) = −0.07
Sit-and-reach (cm)	11.51 ± 5.49	11.91 ± 5.00	0.708	−0.08	*t*(96) = −0.38
Male pull-ups (count)	3.88 ± 3.15	4.08 ± 2.92	0.807	−0.07	*t*(49) = −0.25
Female sit-ups (count)	18.87 ± 6.06	21.00 ± 7.75	0.282	−0.32	*t*(45) = −1.09
Male 1000m (s)	280.76 ± 28.44	280.85 ± 34.11	0.992	−0.01	*t*(49) = −0.003
Female 800m (s)	251.13 ± 34.78	253.79 ± 22.96	0.759	−0.09	*t*(45) = −0.31

### Within-group effects of the intervention

Paired-sample *t*-tests were conducted to examine within-group differences before and after the intervention in both the experimental group (EG) and the control group (CG) (Table 5). In the 50-meter sprint, significant improvements were observed in both the CG (Δ = −0.17 s, 95% CI [−0.32, −0.02], *p* = 0.028, *d* = 0.21) and the EG (Δ = −0.79 s, 95% CI [−0.98, −0.60], *p* < 0.001, *d* = 0.98), with a notably larger effect size in the EG. Similar patterns were found in the standing long jump, where the CG improved by 13.20 cm (95% CI [7.32, 19.08], *p* < 0.001, *d* = 0.35), and the EG by 20.98 cm (95% CI [14.89, 27.07], *p* < 0.001, *d* = 0.56). In flexibility, assessed via the sit-and-reach test, the CG demonstrated a modest improvement (Δ = 1.41 cm, 95% CI [0.45, 2.37], *p* = 0.005, *d* = 0.27), whereas the EG exhibited a more pronounced gain (Δ = 4.55 cm, 95% CI [3.20, 5.90], *p* < 0.001, *d* = 0.82). Upper body strength in male students, measured by pull-ups, significantly increased in both groups (CG: Δ = 1.72 repetitions, 95% CI [0.85, 2.59], *p* < 0.001, *d* = 0.57; EG: Δ = 2.73 repetitions, 95% CI [1.80, 3.66], *p* < 0.001, *d* = 0.82). Among female students, abdominal muscle endurance, measured via 1-minute sit-ups, improved by 1.71 repetitions (95% CI [0.45, 2.97], *p* = 0.009, *d* = 0.27) in the CG and by 3.63 repetitions (95% CI [2.20, 5.06], *p* < 0.001, *d* = 0.55) in the EG. Cardiopulmonary endurance was significantly enhanced in both groups. Among male students, 1000-meter run times decreased significantly in the CG (Δ = −39.36 s, 95% CI [−47.20, −31.52], *p* < 0.001, *d* = 1.32) and the EG (Δ = −35.50 s, 95% CI [−42.30, −28.70], *p* < 0.001, *d* = 1.10). Similarly, female students in the CG improved their 800-meter run times by 18.38 seconds (95% CI [−25.20, −11.56], *p* < 0.001, *d* = 0.62), while those in the EG achieved a reduction of 22.53 seconds (95% CI [−27.30, −17.76], *p* < 0.001, *d* = 0.95). Collectively, these findings suggest that although both groups experienced improvements in physical fitness, the experimental group consistently exhibited greater magnitudes of change, indicating a stronger intervention effect.

**Table 5:** Within-group comparisons of pre-test and post-test measurements in experimental (EG) and control (CG) groups.

Measure	Group	Pre-Test (Mean±SD)	Post-Test (Mean±SD)	Δ [95% CI]	Within p	Cohen’s d	Interpretation
50m Sprint (s)	CG	8.81 ± 0.71	8.64 ± 0.87	−0.17 [−0.32, −0.02]	0.028	0.21	Small effect
	EG	8.83 ± 0.88	8.04 ± 0.77	−0.79 [−0.98, −0.60]	<0.001	0.98	Large effect
Standing long jump (cm)	CG	208.46 ± 37.54	221.66 ± 37.68	13.20 [7.32, 19.08]	<0.001	0.35	Small effect
	EG	208.96 ± 37.35	229.94 ± 39.78	20.98 [14.89, 27.07]	<0.001	0.56	Medium effect
Sit-and-reach (cm)	CG	11.51 ± 5.49	12.92 ± 5.13	1.41 [0.45, 2.37]	0.005	0.27	Small effect
	EG	11.91 ± 5.00	16.46 ± 6.51	4.55 [3.20, 5.90]	<0.001	0.82	Large effect
Male Pull-ups (n)	CG	3.88 ± 3.15	5.60 ± 2.94	1.72 [0.85, 2.59]	<0.001	0.57	Medium effect
	EG	4.08 ± 2.92	6.81 ± 3.93	2.73 [1.80, 3.66]	<0.001	0.82	Large effect
Female Sit-ups (n)	CG	18.87 ± 6.06	20.58 ± 6.92	1.71 [0.45, 2.97]	0.009	0.27	Small effect
	EG	21.00 ± 7.75	24.63 ± 5.24	3.63 [2.20, 5.06]	<0.001	0.55	Medium effect
Male 1000m (s)	CG	280.76 ± 28.44	241.40 ± 31.76	−39.36 [−47.20, −31.52]	<0.001	1.32	Very large effect
	EG	280.85 ± 34.11	245.35 ± 25.43	−35.50 [−42.30, −28.70]	<0.001	1.10	Large effect
Female 800m (s)	CG	251.13 ± 34.78	232.75 ± 23.32	−18.38 [−25.20, −11.56]	<0.001	0.62	Medium effect
	EG	253.79 ± 22.96	231.26 ± 24.09	−22.53 [−27.30, −17.76]	<0.001	0.95	Large effect

Notes. Δ [95% CI]: Mean difference (post-test minus pre-test) with 95% confidence intervals (units match original metrics). Within p: Significance from paired t-tests (*p < 0.05* considered significant). Cohen’s d: Effect size thresholds: small (≥0.2), medium (≥0.5), large (≥0.8). Assumptions: All pre-post differences passed Shapiro-Wilk normality tests (*p > 0.05*).

### Between-group effects of the intervention

After controlling for baseline scores using ANCOVA, significant between-group differences were observed in multiple physical fitness indicators (Table 6). Specifically, the experimental group (EG) exhibited greater improvements in 50-meter sprint performance compared to the control group (CG), with a mean difference of ΔΔ = –0.62 seconds, 95% CI [–0.85, –0.39], *p* < 0.001, partial η^2^ = 0.18, Cohen’s *d* = 0.75, indicating a large effect size. Similarly, EG demonstrated superior gains in standing long jump performance (ΔΔ = 7.78 cm, *p* = 0.022, η^2^ = 0.05, *d* = 0.41) and sit-and-reach flexibility (ΔΔ = 3.14 cm, *p* < 0.001, η^2^ = 0.12, *d* = 0.60), reflecting medium to large effects. Among male students, pull-up performance increased significantly in the EG (ΔΔ = 1.01 repetitions, *p* = 0.030, η^2^ = 0.05, *d* = 0.41), while among female students, significant improvements were observed in the 1-minute sit-up test (ΔΔ = 1.92 repetitions, *p* = 0.029, η^2^ = 0.06, *d* = 0.45). However, no statistically significant between-group differences were found in male students’ 1000-meter running times (ΔΔ = 3.86 seconds, *p* = 0.400, η^2^ = 0.01, *d* = 0.12) or female students’ 800-meter running times (ΔΔ = –4.15 seconds, *p* = 0.178, η^2^ = 0.03, *d* = 0.18). These findings suggest that the FRTSA intervention produced significant and meaningful improvements in students’ speed, power, flexibility, and muscular strength, particularly in short-duration, skill-related physical fitness domains.

**Table 6:** ANCOVA-based between-group comparison of intervention effects.

Measure	ΔΔ [95% CI]	ANCOVA p	η^2^	Between d	Interpretation
50m Sprint (s)	−0.62 [−0.85, −0.39]	<0.001	0.18	0.75	EG improved significantly more (large effect).
Standing LJ (cm)	7.78 [1.12, 14.44]	0.022	0.05	0.41	EG showed greater jump distance gains (medium effect).
Sit-and-reach (cm)	3.14 [1.50, 4.78]	<0.001	0.12	0.60	EG flexibility improved markedly (large effect).
Male Pull-ups (n)	1.01 [0.10, 1.92]	0.030	0.05	0.41	EG strength gains exceeded CG (medium effect).
Female Sit-ups (n)	1.92 [0.20, 3.64]	0.029	0.06	0.45	EG core endurance improved more (medium effect).
Male 1000m (s)	3.86 [−5.20, 12.92]	0.400	0.01	0.12	No significant between-group difference.
Female 800m (s)	−4.15 [−10.20, 1.90]	0.178	0.03	0.18	No significant between-group difference.

Notes. ΔΔ [95% CI]: Adjusted mean difference of improvements (ΔEG − ΔCG) with 95% CIs. ANCOVA p: Significance after controlling for pre-test values. *η*^2^: Partial eta-squared effect size: small (≥0.01), medium (≥0.06), large (≥0.14). Between d: Standardized mean difference (post-test) using pooled SD. Limitations: Potential confounders (e.g., training history, diet) were not controlled.

While both the FRTSA and control groups demonstrated significant within-group improvements across all physical fitness domains—reflecting natural maturation, seasonal training effects, or routine physical activity—the magnitude and significance of gains differed meaningfully between groups. ANCOVA analyses revealed that the FRTSA intervention produced significantly greater improvements in speed (50m sprint), explosive power (standing long jump), flexibility (sit-and-reach), and muscular endurance (pull-ups/sit-ups), but not in aerobic endurance (1000-m/800-m run), highlighting its particular efficacy in skill- and strength-related physical fitness domains.

## Discussion

This quasi-experimental study provides compelling evidence that a 16-week Feature Recess-Time Sports Activities (FRTSA) protocol significantly enhances multiple domains of physical fitness (PF) among rural Chinese high school students, outperforming traditional Recess-Time Sports Activities (RTSA). The findings support both initial hypotheses: (1) participants undergoing FRTSA demonstrated significant within-group improvements in PF measures, and (2) the FRTSA group (EG) achieved superior post-intervention outcomes compared to the control group (CG).

Our results revealed that students in the experimental group exhibited significantly greater improvements in short-term, skill-related fitness indicators—particularly 50-meter sprint time, standing long jump distance, sit-and-reach flexibility, and gender-specific muscular strength tests—relative to their peers in the control group. These between-group differences emerged after adjusting for baseline equivalence, as no significant differences were observed between groups at the pre-test.

The largest effect sizes were observed in the 50-meter sprint (Cohen’s d = 0.75) and sit-and-reach test (Cohen’s d = 0.60). These findings align with prior literature suggesting that repetitive exposure to functional, rhythmic activities such as rope skipping and structured gymnastics enhances neuromuscular coordination and lower-limb explosiveness [39–40]. Moreover, the yoga-based relaxation sessions, which emphasized static stretching and controlled breathing, likely contributed to the gains in flexibility. Such mind–body practices have increasingly been linked to improved joint range of motion and proprioceptive awareness in adolescents [41–42].

Moderate but statistically significant improvements were also observed in strength measures—pull-ups for males (Cohen’s d = 0.41) and 1-minute sit-ups for females (Cohen’s d = 0.45). These results reinforce the effectiveness of bodyweight-based, repetitive movement formats—particularly rope skipping and dynamic gymnastics—in developing muscular endurance during adolescence. These findings are in line with Rico-González [43], who emphasized that structured and playful strength-based activities are developmentally appropriate and efficacious in school-based physical education.

In contrast, the intervention did not produce statistically significant between-group differences in 1000-meter (males) and 800-meter (females) endurance runs. While the serpentine running activity was designed to enhance aerobic capacity, it may not have provided sufficient volume, intensity, or specificity to exceed the performance gains elicited by the traditional long-distance running adopted in the control condition. This may reflect the principle of specificity, as the CG’s intervention was more closely aligned with the task demands of the aerobic endurance assessments.

These findings suggest that while FRTSA is effective in improving components such as speed, strength, flexibility, and coordination, its current aerobic training component may require further development. Future iterations of the FRTSA model may benefit from integrating higher-intensity or longer-duration aerobic elements—such as interval running or circuit-based endurance drills—to better target cardiorespiratory fitness.

Overall, this study highlights the value of a multi-modal, scaffolded, and engaging physical activity framework—such as FRTSA—in enhancing adolescent physical fitness. Compared to the monotonous collective running utilized in the RTSA group, the diversity and structure of FRTSA activities may not only promote broader physical performance gains but also foster greater intrinsic motivation and participation adherence. These behavioral factors are essential for long-term physical activity engagement and health promotion in youth [21,44–46]. Future research should explore longitudinal follow-ups to assess whether the observed fitness improvements are sustained over time, as well as examine potential mediating effects of psychological outcomes (e.g., motivation, enjoyment) associated with the FRTSA model. Additionally, further refinement of the intervention’s aerobic component could enhance its comprehensiveness and impact.

A limitation of this study was that outcome assessors were not blinded to group allocation. Although standardized protocols and objective instruments (e.g., electronic timers) were used for key tests such as the 50-meter sprint and endurance runs, performance assessment for sit-ups and jump technique relied on direct observation, which may have introduced subtle measurement or performance biases. Future studies should consider involving independent evaluators or video-based scoring to minimize observer expectancy effects.

Additionally, several potential confounding factors—such as dietary intake, participation in extracurricular physical activities, and socioeconomic status—were not measured in this study. While randomization helps distribute such variables evenly across groups, residual confounding cannot be ruled out, particularly in rural settings where access to nutrition and out-of-school sports may vary significantly. These factors could influence baseline fitness levels and responsiveness to intervention.

The observed effects may also be shaped by behavioral and environmental mechanisms. The FRTSA program’s structured yet playful design likely enhanced intrinsic motivation and adherence compared to the repetitive, drill-based nature of the control group’ s military-style running. In rural Chinese schools, where extracurricular physical activity opportunities are often limited, the innovative and diversified structure of FRTSA may have provided a unique stimulus for engagement [47]. Furthermore, the integration of music, peer interaction, and progressive skill development aligns with self-determination theory, supporting autonomy, competence, and relatedness—key drivers of sustained physical activity in adolescents [45–46]. These psychosocial factors, while not directly measured, may partially explain the superior improvements in speed, strength, and flexibility observed in the intervention group.

## Conclusions

In conclusion, FRTSA protocol demonstrates substantial potential as a developmentally appropriate, inclusive, and pedagogically grounded intervention for promoting multidimensional physical fitness among adolescents. Its capacity to elicit significant and clinically meaningful improvements—particularly in speed, explosive power, and flexibility—underscores its alignment with critical neuromotor development objectives during early adolescence. These findings support the integration of FRTSA into school-based physical activity programs as a feasible, scalable, and sustainable approach to enhancing health-related fitness outcomes. Future research should investigate the long-term sustainability of these benefits and examine the applicability and effectiveness of this model across diverse educational and demographic contexts.

## Supporting information

S1 Appendix Physical health measurement score. (DOCX)
