# Supplementary material for: Feature recess-time sports activities as a school-based intervention to improve fitness in rural Chinese youth
Source: PLoS One. 2026 Jan 22;21(1):e0337716. doi: 10.1371/journal.pone.0337716 (PMC12826519; doi:10.1371/journal.pone.0337716)
Supplement: S1 Appendix — (DOCX) [file pone.0337716.s001.docx]

**Physical Health Measurement Score**

| **Number** | **Gender** | **Age** | **Body Shape** | | | | | **Physical Function** | **Note** |  |
| --- | --- | --- | --- | --- | --- | --- | --- | --- | --- | --- |
|  |  |  | **Height**  **(cm)** | **Weight**  **(kg)** | **BMI (%)** | | | **vital capacity(ml)** |  |  |
|  |  |  |  |  |  | | |  |  |  |
|  |  |  |  |  |  | | |  |  |  |
|  |  |  |  |  |  | | |  |  |  |
|  |  |  |  |  |  | | |  |  |  |
|  |  |  |  |  |  | | |  |  |  |
|  |  |  |  |  |  | | |  |  |  |
|  |  |  |  |  |  | | |  |  |  |
|  |  |  | **Physical Quality** | | | | | |  |  |
| **Number** | **Gender** | **Age** | **Speed**  **(s)** | **Endurance**  **(min. s)** | | **Strength**  **（pcs）** | | **Explosive**  **（m）** | **Flexible**  **（cm）** |  |
|  |  |  | 50m | Male: 1000m | Female: 800m | Male:  pull up | Female:   sit ups | Standing long jump | Sit and reach |  |
|  |  |  |  |  |  |  |  |  |  |  |
|  |  |  |  |  |  |  |  |  |  |  |
|  |  |  |  |  |  |  |  |  |  |  |
|  |  |  |  |  |  |  |  |  |  |  |
|  |  |  |  |  |  |  |  |  |  |  |
|  |  |  |  |  |  |  |  |  |  |  |

Note: Anthropometric and physical fitness assessments were conducted, following CNSPFS, 2014 revision. Standardized tests included:

**Speed:** 50-m sprint

**Explosive Power**: Standing long jump

**Flexibility**: Sit-and-reach test

**Strength**: Pull-ups (males) / 1-min sit-ups (females)

**Endurance**: 1000-m run (males) / 800-m run (females)
